# Supplementary figures and images for: Attenuation of HIV severity by slightly deleterious mutations can explain the long-term trajectory of virulence evolution
Source: PLoS Comput Biol. 2025 Dec 1;21(12):e1013131. doi: 10.1371/journal.pcbi.1013131 (PMC12677797; doi:10.1371/journal.pcbi.1013131)

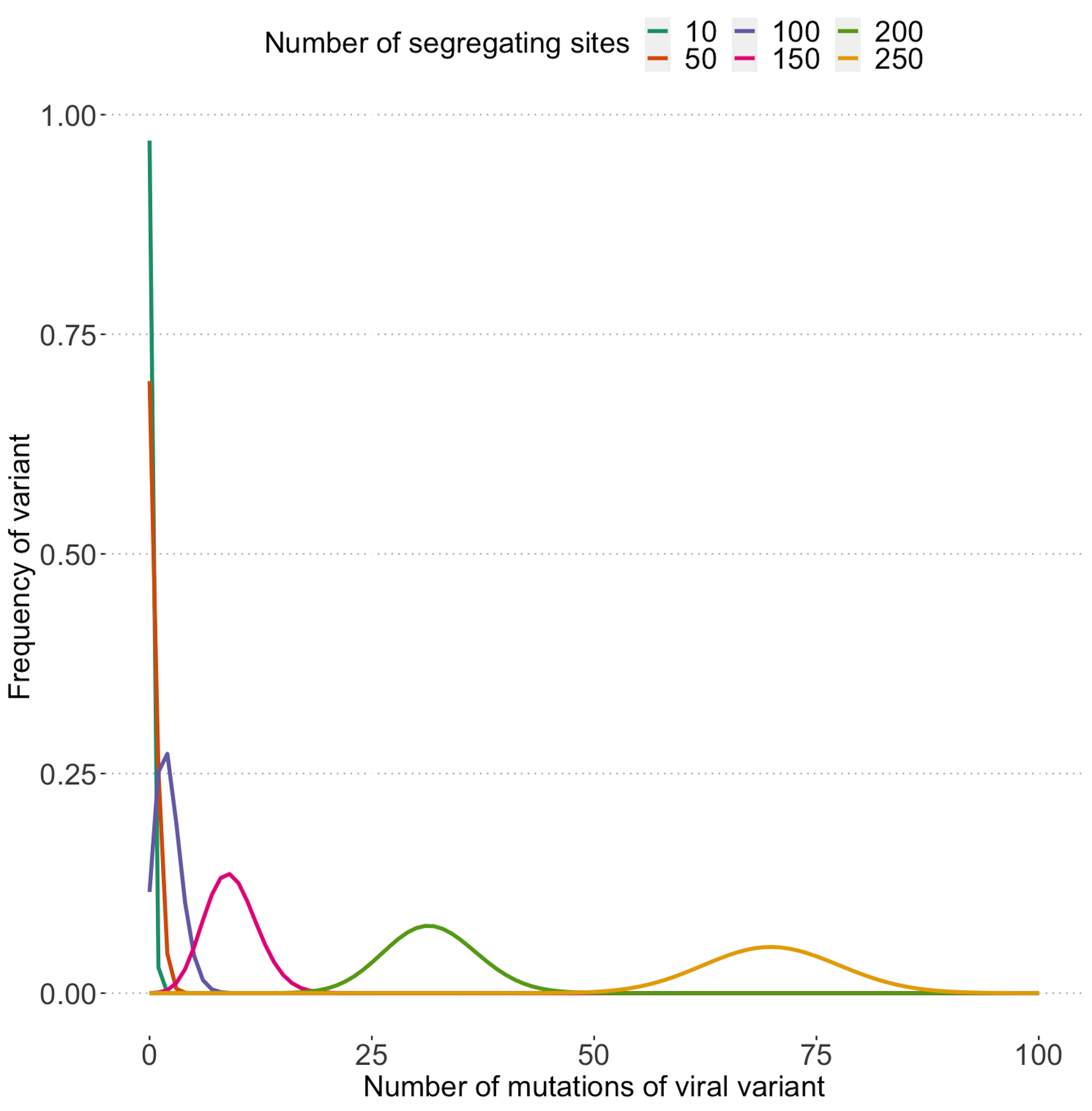

Supplement: S1 Fig — A specific viral type is defined by its number of mutations. When we consider few mutations of large effect, the population is dominated by a single virus type of high relative fitness. As we increase the number of segregating sites and lower the associated fitness cost, the population becomes increasingly diverse, which has implications for the viral variants that are transmitted and between-host evolution. This diversity in the within-host viral population creates a broader pool of viral variants available for transmission, potentially influencing both transmission dynamics and the trajectory of between-host evolution. (TIFF) [file pcbi.1013131.s001.tiff]

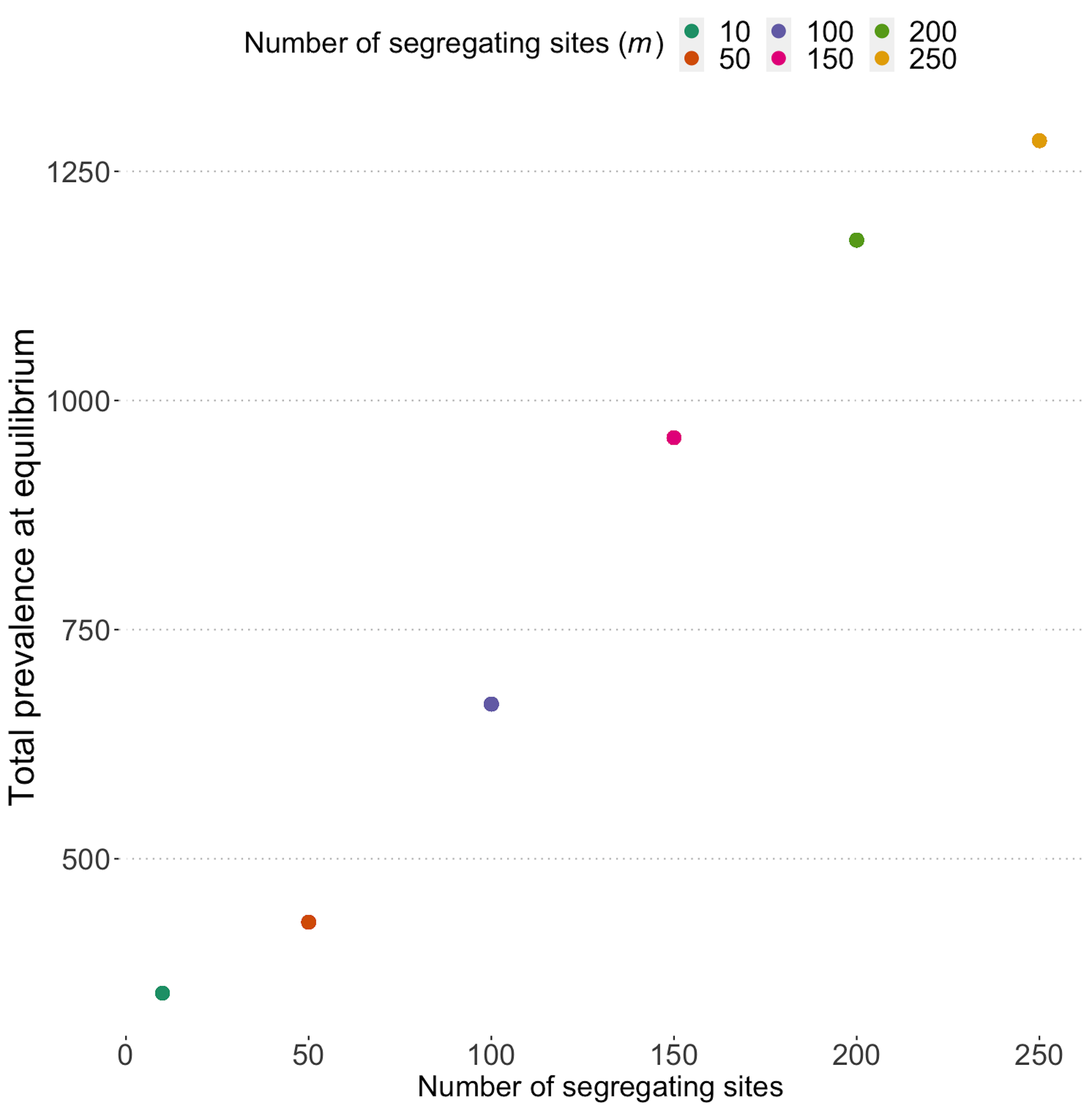

Supplement: S3 Fig — As the number of segregating sites increases, the within-host dynamics slow down and the viral population is more diverse. As a result, between-host selection is able to select the virus types with greatest transmission potential, ultimately increasing the endemic prevalence. (TIFF) [file pcbi.1013131.s003.tiff]
