# Supplementary material for: Attenuation of HIV severity by slightly deleterious mutations can explain the long-term trajectory of virulence evolution
Source: PLoS Comput Biol. 2025 Dec 1;21(12):e1013131. doi: 10.1371/journal.pcbi.1013131 (PMC12677797; doi:10.1371/journal.pcbi.1013131)
Supplement: Attachment — Submitted filename: Response to reviewers.pdf [file pcbi.1013131.s005.pdf]

**Response to reviewers**

We are very grateful for the insightful and constructive feedback from both reviewers and thank them for their careful review of our work. In light of their comments we have made several changes to the manuscript, outlined below. The major changes have been highlighted in red text in the revised manuscript.

Reviewer #1: In their paper "Attenuation of HIV severity by slightly deleterious mutations can explain the long-term
trajectory of virulence evolution", Longley et al propose a new explanation for the lack of specific mutations that are associated ith set-point viral loads. According to their hypothesis many slightly deleterious mutations could be responsible for that. This could also explain the wide variation is set-point viral loads across infected individuals.

This paper presents an original and appealing hypothesis for some of the remaining discrepancies in our understanding of the evolution of the set-point virus load of HIV. To assess its validity, the authors developed a computational model that takes into account the within-host dynamics, diversification and selection of HIV, and its epidemiological transmission. While the resulting model is fairly complex, it is built on previous modeling work (by Lythgoe, Pellis and Fraser) and its parameters are empirically well supported. I have been reviewing this work a few weeks ago in the context of the viva of the first author. I already liked it the first time I read it: an inspiring conceptual idea assessed by well-executed modeling and analysis. I therefore have only minor points to raise.

My first point relates to the motivation of the study: in short the authors say that the heritability of the set-point viral load is between 20-30% (l.63), but the genetic basis for this remains unclear because:
"Specific viral mutations with large effects on fitness have been identified (44), but these are relatively low in number and the total narrow-sense heritability from all genetic hits is significantly lower than our estimates of broad-sense heritability." (l.520)
Ref 44 - Gabrielaite et al - estimates that four viral mutations account for 8.2% of the variation in the set-point viral load. While this does not add up to 20%, it is consistent with broad-sense heritability estimates by Hodcroft et al (5.7% - not cited by the authors) and Bertels et al (8% - cited) that assumed neutral (Brownian) trait evolution. (In my view, these estimates were lower than previous estimates because they were adjusted for covariates such as age and sex.) Assuming stabilizing ("Ornstein-Uhlenbeck") selection, the heritability estimates are higher (e.g. 29% in Bertels et al), and this type of selection has more statistical support than neutral evolution of the set-point virus load.

It would be great if the authors could discuss briefly if the narrow-sense heritabilities estimated by GWAS should be compared to the estimates based on neutral or stabilizing trait evolution. GWAS usually adjust for demographic covariates, so their heritability estimates should be compared to adjusted broad-sense estimates. But I am unclear about the assumptions about trait evolution underlying GWAS. This is important as it would tell us exact how far off the different heritability estimates are.

We thank the reviewer for this thoughtful and detailed comment. We agree that comparing narrow-sense heritability estimates from GWAS to broad-sense estimates derived from phylogenetic models requires careful consideration of both the evolutionary assumptions and covariate adjustments underlying each approach.

In response, we have revised the introduction to more clearly explain these distinctions. Specifically, we now highlight that:

- Broad-sense heritability estimates vary depending on whether neutral (Brownian motion) or stabilizing (Ornstein–Uhlenbeck) models are assumed.
- Lower estimates (e.g., 5.7% in Hodcroft et al. and 8% in Bertels et al.) are consistent with neutral models, while stabilizing selection yields higher estimates (e.g., 29% in Bertels et al.).
- GWAS typically estimate narrow-sense heritability, adjusted for demographic covariates, and reflect only the additive genetic variance explained by identified variants.

We have also clarified that GWAS estimates are most reasonably compared with covariate-adjusted broad-sense estimates, though they are not directly equivalent due to differences in the types of genetic effects captured and methodological assumptions.

It would be great if the authors could specify the mutation matrix qij. They state on l.133 that "each newly infected cell can acquire or lose at most one mutation". Does that mean that going from the reference with no mutation to a single mutation happens with the genomic mutation rate 3x10^-5*10,000 = 0.3 while loosing that specific mutation happens with 3x10^-5*m? (What confuses me is that there is m single-point mutants in class x_1, choose(m,2) in x_2, etc, so forward and backward mutation rates between virus type i and j are different, vary by i, and depend on the number of segregating sites.)

We are only looking at m sites and so the entries of $Q$ are defined as:

1. Forward mutation (type $j \rightarrow j+1$): $q_{j+1,j} = \mu \cdot (m-j)$
2. Backward mutation (type $j \rightarrow j-1$): $q_{j+1,j} = \mu \cdot j$
3. No mutation (type $j \rightarrow j$): $q_{j+1,j} = 1 - \mu \cdot m$

This clarification has been added to the methods section.

The authors simulate with varying numbers of segregating sites from 10 to 250 and use the landscape of mutation costs estimated by Zanini et al (ref 36). Could the authors state how many segregating site Zanini et al observed in their data and the average cost they found for comparison with the authors' modeling assumptions? (It could even be plotted into Fig. 1A.)

The maximum number of sites we could feasibly model was 250, however the number of segregating sites is substantially larger than this, as shown in Zanini et al. The following paragraph has been added to the discussion expand on this point:

*A study of the fitness landscape of the HIV genome showed that for a substantial fraction of the genome mutations are weakly deleterious (cost <1%), particularly at synonymous sites but also at non-synonymous sites (38). Synonymous mutations, while not directly affecting protein structure, can still impede viral fitness by influencing RNA stability, translation efficiency, or protein folding. The study also highlighted evidence that the deleterious component of the landscape is universal across infections and is fundamental to the high diversity within HIV group M diversity. In our modelling framework, it was not possible to explore beyond 250 segregating sites due to the significant computational demand of tracking within-host and between-host dynamics. While this represents only a subset of the possible mutational landscape, our qualitative findings are expected to hold if a larger number of sites were included. The scenarios explored here serve as a proof of principle, demonstrating how the accumulation of many weakly deleterious mutations can shape viral load distributions and evolutionary outcomes at both scales.*

Lastly, it would be great if the authors could comment on how CTL escape mutations, that often occur during HIV infection and have large beneficial fitness effects, would affect their results. The authors discuss how the HLA alleles of hosts might affect the set-point virus load evolution. Would this render deleterious mutations less effective in slowing within-host evolution, or the reverse? (I am aware that the authors discuss van Dorp et al's work provides an alternative hypothesis for the evolution of the set-point viral load of HIV. I am basically asking what happens if you combine the two hypotheses.)

We thank the reviewer for this thoughtful and important comment. We agree that CTL escape mutations, driven by host-specific HLA-mediated immune pressure, represent a key mechanism in HIV evolution and could interact with the processes described in our model.

In response, we have added a paragraph to the Discussion section that explicitly considers how CTL escape dynamics might influence the evolution of spVL, especially in combination with deleterious mutation accumulation. We emphasise that while CTL escape mutations can offer short-term fitness advantages within hosts, they often carry costs that reduce fitness upon transmission to hosts with mismatched HLA types. These dynamics are well-documented (e.g., in the form of HLA footprints), but their long-term impact on population-level viral fitness is constrained by the high diversity of HLA alleles across hosts.

Importantly, we now explicitly note that it is difficult to predict the precise effects of incorporating CTL escape mutations into our model, as the outcome would depend on multiple interacting factors—including the timing and frequency of escape events, the fitness costs of escape mutations, the degree of HLA matching between transmission pairs, and the potential for compensatory mutations. While such complexity could increase heterogeneity in viral evolution across hosts and potentially modulate the strength of selection against deleterious mutations, we argue that the general mechanism of mutation accumulation remains a robust and parsimonious explanation for the observed evolutionary patterns in spVL.

Very minor points and typos:
- Fig. 1,2,3,4,5 do not require color

These plots have been changed to black.
- there are still instances of "I" instead of "we", e.g. "I applied" in l. 495.

This has been corrected

Reviewer #2: Plos Comp Bio June 2025

I love the main idea of this paper, to try to determine if many mutations of small deleterious effect could explain the variation in viral loads and the heritability of viral loads in HIV.

I enjoyed thinking about the model you proposed. I imagine that a large number of sites where mutations are slightly deleterious creates genetic variation within the host (quasi species or mutation selection balance). The resulting viral load influences the length of infection and also infectivity. I guess you are suggesting that some deleterious mutations and therefore lower VL would be good for the virus in the long run because it lengthens the live of the host and therefore increases the chance of infecting a new host. Then, from the within-host variation, a random viral particle infects the new host. If within host variation is very large, then heritability will be low, but if within host variation is moderate, we could get some heritability and also between host variation.

In addition, to keep some between host variation, you need the dynamics of reaching the mutation-selection equilibrium to be slow (if fast, every host would have the same VL within a year). To get slow dynamics, you need small selection coefficients. To get within and between host variation you need a high number of sites that can mutate. I think that your model would also work if you added a range of selection coefficients for the sites. There is no need to have only one s value.

We thank the reviewer for their insightful comments, and for clearly articulating the dynamics underlying our model. We agree that introducing a distribution of selection coefficients (*s*) could be a valuable extension. However, our primary aim was to test whether the accumulation of *many* mutations with *small* deleterious effects can explain the observed variation and heritability in SPVL.

To isolate this hypothesis, we fixed *s* to focus specifically on the regime of weak selection across many sites. The absolute value of *s* is less important than the overall effect: whether cumulative, weakly deleterious mutations can generate substantial variation in viral load and heritability across hosts.

We did not explore scenarios involving *many* large-effect mutations or *few* small-effect mutations, as these would not produce a comparable cumulative impact on viral load. In the case of many large-effect mutations, strong purifying selection would act quickly to remove them, limiting standing variation. Conversely, a small number of weak-effect mutations would not be sufficient to influence viral load meaningfully. Our focus on many small-effect mutations is therefore essential to the logic of the model, as it allows us to generate comparable viral load distributions across different parameterisations.

While I think the paper is interesting and could be important for the field, I have some serious concerns.

1. I don't like that you vary the number of sites where mutations can occur (m) with the selection coefficient of the mutations (s), as shown in figure 1A. [By the way, fig 1A should maybe be in the methods? It is an assumption, not a result).
The reason I don't like it is because it doesn't allow you to see the effect of m and s separately. The selection coefficient s should determine the time it takes until an equilibrium is reached. The number of mutations m should determine the average fitness of the population at equilibrium.
I would love to see a series of heatmaps (or similar) that show values of m on the x-axis, values of s on the y-axis and then on the z-axis the average VL, the between host variation in VL, and the heritability of VL.

We thank the reviewer for this thoughtful suggestion. We agree that disentangling the effects of *m* and *s* could yield further insights into the model's behaviour, particularly in terms of equilibrium dynamics and population-level variation.

As noted above, we chose to vary *m* inversely with *s* to ensure a comparable minimum viral load across different parameter regimes, thereby allowing us to isolate the effects of mutation accumulation on between-host variation and heritability. However, we acknowledge that this coupling limits the ability to assess the individual contributions of *m* and *s*.

Unfortunately, the high dimensionality and memory requirements of the model—due to the nested within- and between-host dynamics and the large matrices needed to track virus types—make it computationally infeasible to simulate the full grid of *m* and *s* values needed to generate heatmaps, Nonetheless, to partially address this concern, we have added a new supplementary figure showing equilibrium results with fixed *m* = *100* and varying *s*, which illustrates how increasing *s* results in faster convergence to mutation-selection balance and higher average population fitness.

2. Your result in figure 1B, that more mutations lead to lower fitness, independent of the selection coefficient is a well known result in population genetics. See for example equations 1.18 - 1.20 in Joachim Permissions pop gen notes: https://www.mabs.at/fileadmin/user_upload/p_mabs/2024-lecturenotes.pdf

[You can find this in many books too, but these notes are free to access].
It would be good if you mention the term “mutational load” which is what population geneticists call this effect. It’d also be good to check if your model still has this behavior if you change s but not m.

Thank you for pointing out the link with a pop gen result, we have included this in the relevant results section of the paper. As mentioned above, we have also added a supplementary result for m=150 and s varying, showing that this both changes the tempo but also the average viral load at mutation-selection balance.

3. Somewhere in the results, you decide that m = 250 is the best fitting parameter choice. I am not entirely convinced by that, because it seems to me that this depends a lot on your choice of s and your choice of the max VL.

We do not argue that m=250 is the best fitting parameter, instead it was the limit of our computational capacity. To make this clearer in the text, we have added a section to the discussion regarding the maximum number of sites included, as also described in response to reviewer 1.

4. I am not sure I am convinced of adding a host effect to explain the variation in VL among hosts. I guess you need to find parameter values that lead to enough variation between hosts and also sufficient heritability. I guess your paper is meant to be a proof of concept, and not a formal fitting of the model to available data, but maybe you can explain your choices a little more here. Currently it feels a little ad hoc.

We appreciate the reviewer's comment and agree that the addition of a host effect could appear ad hoc without further explanation. Our motivation for including this component was to provide a simple, tractable way to account for host-specific factors—particularly HLA-related immune pressure—that are known to contribute to variation in SPVL but are not explicitly modelled in our framework.

Rather than building a full model of HLA diversity or CTL escape dynamics (which would add considerable complexity and require extensive immunological parameterisation), we opted to include a fixed host effect as a stand-in for this variation. This approach allowed us to assess how host-level variance, alongside viral genetic factors, might influence observed patterns of spVL heritability.

We agree that this is a simplified representation, and we now clarify this point more explicitly in the manuscript. We have also added a discussion of how future models could integrate host genetic diversity more mechanistically, and how this could help refine the interpretation of viral heritability estimates. We hope this helps to frame our model as a proof of concept designed to test a specific hypothesis, while highlighting key directions for future work.

5. Would it be possible to show some zoomed in results from your simulations? Like for a given host, what is the behavior of individual mutations? How does the VL behave in that host? This is just out of curiosity & to get a better sense of what is happening at the host level and how that influences the epidemic level?

While this is an interesting point, we focus on the number of mutations and do not track specific mutations in our analysis, and therefore with the current framework this would not be possible.

Small things:

Line 30-32: This statement seems a little strong in my opinion because only one (or two) paper have shown this.

We have removed the word 'prominent' and changed this just to 'is an example'

Line 34-36: I don't understand this sentence.

Sentence has been rephrased.

Line 41: trade-offs. I think there is a new review on trade offs out of Brandon Ogbunu's lab. May be worth citing here. Also: here you argue that it is hard to proof a trade-off (I agree) which contradicts with the strong statement in the first sentence of the Introduction).

Line 64: "Most part unexplained" — this makes me curious, which part *is* explained?

This paragraph has been rewritten.

Line 69-71: I don't understand this sentence.

This paragraph has been rewritten.

Line 82-94: I found this paragraph somewhat hard to follow.

Line 111: "segregating sites" This was confusing to me because they are potentially segregating, but not necessarily, right? Maybe just say: sites.

Changed to sites.

Line 121: the quasi species equation is the same as mutation-selection balance, right?

Yes, the relevant methods section has been rephrased to make this clearer.

Line 136: something odd with a footnote at the end of the line.

Line 153: I don't understand why you need to track all possible combinations of viral variants. Not a big deal, just curious.

This means that for a virus type i (starts with i mutations) we need to know the proportion in the within host population of every virus type j (1, ..,m) at each time point.

Line 181: lambda = 5 / m . Why 5? I am confused!

This is how we fix the viral load range to be 2-7 log10

Line 194: hill should be Hill

Line 283: "I" should be "we"?

Line 303: "effectively neutral" I don't understand that statement.

We have changed to be ‘very close to zero’

Line 360: “equilibrium is reached” I don’t agree with that. The figures don’t look like an equilibrium is reached.

Clarified this to emphasise that the equilibrium is reached after the 20 year maximum infection period, hence why it isn’t seen on the figure

Line 374: “next generation framework” I am not sure what that means here.

Line 380: here you say that increased m leads to slower dynamics, but it is actually decreased s that does this.

Sentence has been rewritten to link this dynamic change with s.

Figure 4: I suggest using yellows for this figure to show that it is linked to the m = 250 case of the previous figure.

Line 437: “Short sighted” I am not sure why this is considered short sighted. Is it because it doesn’t maximize infectivity? Now that I am typing this up, I guess I am not sure how infectivity is affected in your model by m and s.

Higher viral load -> higher probability of transmission
